# A case report of long treatment with Itraconazole in a patient with chronic Chagas disease

**DOI:** 10.1186/s12879-019-4608-9

**Published:** 2019-11-09

**Authors:** Pau Bosch-Nicolau, Fernando Salvador, Adrián Sánchez-Montalvá, Elena Sulleiro, Joaquín Burgos, Israel Molina

**Affiliations:** 1 Department of Infectious Diseases, Vall d’Hebrón, University Hospital, Pº Vall d’Hebrón 119-129, 08035 Barcelona, Spain; 2 Department of Medicine, Universitat Autònoma de Barcelona, Barcelona, Spain; 3 Department of Microbiology, Vall d’Hebron, University Hospital, PROSICS Barcelona, Barcelona, Spain

**Keywords:** Chagas disease, Ergosterol synthesis inhibitors, *Trypanosoma cruzi*

## Abstract

**Background:**

Current available treatments (benznidazole and nifurtimox) for Chagas disease (CD) show limited efficacy in chronic phase and frequent undesirable effects. Ergosterol synthesis inhibitors (ESI) had been considered as promising drugs for CD treatment and despite its recent poor results in several clinical trials, different strategies have been proposed to optimize its role in this infection.

**Case presentation:**

We present a case of chronic Chagas disease in patient diagnosed with HIV who received treatment for histoplasmosis with itraconazol during twelve months. Even though *T. cruzi* rt-PCR was persistently negative during treatment, when itraconazol was stopped she presented with a positive blood rt-PCR.

**Conclusion:**

Several studies using different ESI had been published for CD treatment. Either in vitro or in vivo assays demonstrated activity against *T. cruzi* of the different triazole derivatives so different clinical trials had been carried out to evaluate its efficacy and safety. Despite contradictory evidence in the animal model, longer treatments along with other treatment strategies previously proposed suggests that ESI failure rates in positive peripheral blood rt-PCR are higher than that obtained with the current treatments of choice.

## Background

Despite of the growing consensus of treating Chagas disease (CD) in chronic stages to avoid the progression of chagasic cardiomyopathy, current available treatments (benznidazole and nifurtimox) show limited efficacy reaching cure rates between 15 and 35% [1] and frequent undesirable effects, which leads to definitive withdrawal of the treatment in 13–32% of patients [2]. Triazole derivatives (ketoconazole, itraconazole, posaconazole, voriconazole, ravuconazole) have been used for years as antifungal treatments with a good pharmacokinetic and safety profile. They act as selective inhibitors of *T. cruzi* ergosterol synthesis showing potent intrinsic activity against the parasite in both in-vitro and in-vivo models, and had been considered as promising drugs for CD treatment [3]. Different strategies have been proposed to optimize its outcomes in CD. Here we present a treatment failure, after the longest treatment with ergosterol synthesis inhibitors (ESI) reported in a patient with chronic CD.

## Case presentation

**Fig. 1:**
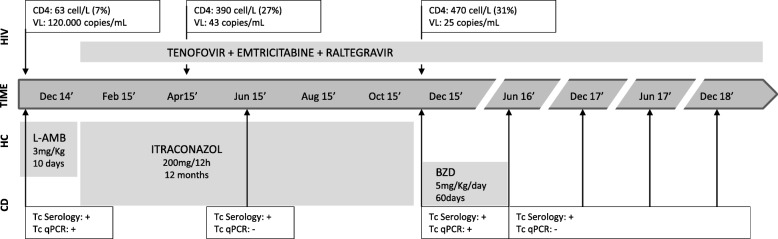
Timeline of case presentation Description: Timeline describing patient evolution regarding HIV, Histoplasma and Chagas disease. Legends: BZD: Benznidazole, CD: Chagas disease, CD4: CD4+ Leucocytes T count, HC: Histoplasmosis, HIV: Human immunodeficiency virus, L-AMB: Liposamal Amphotericine B, qPCR: real-time quantitative polymerase-chain-reaction, Tc: *Trypanosoma cruzi*, VL: Viral load

A 38-Year-Old woman presented with a 2 months history of abdominal pain, weight loss and constipation. She was born in Bolivia (Sucre), and moved to Barcelona 7 years ago. She had no relevant pathological history. A Computed tomography (CT) showed an ileal stenosis causing intestinal occlusion. Considering a differential diagnosis including malignancies, inflammatory disease or infectious diseases an ileum resection with termino-terminal anastomosis was carried out. The biopsy revealed the presence of a granulomatous ileitis with a PAS and Gomori stain showing fungal microorganisms and a real-time quantitative polymerase-chain-reaction (qPCR) with a positive result for *Histoplasma spp*. An HIV serology was done showing an advanced HIV infection with a CD4 count of 63 cell/L (7%) and a viral load of 120,000 copies/mL. Due to the fact that was the first contact to health system a screening for imported diseases was offered including hepatitis B and C, *Treponema pallidum*, *Strongyloides stercoralis* and *Trypanosoma cruzi* serology and investigation for parasites in feces. Two positive serologic tests were obtained for *T. cruzi*: one of them using recombinant antigen (Bioelisa Chagas, Biokit, Lliçà d’ Amunt, Spain) and the other one using a crude antigen (*T. cruzi* ELISA, Ortho-Clinical Diagnostics, Johnson & Johnson, High Wycombe, United Kingdom). Cardiac and gastrointestinal involvement was assessed by a 12-lead electrocardiography, chest radiography, an echocardiography, a barium enema examination, and an esophagogram showing no visceral involvement. We started antiretroviral therapy with Tenofovir/Emtricitabine and Raltegravir with rapid improve of CD4 count to normal values within 6 months. Histoplasmosis was treated with liposomal amphotericin 3 mg/kg during 10 days followed by itraconazole 200 mg/12 h during 12 months with monthly visits at the outpatient clinic assessing adherence to treatment and adverse effects. After 6 months of ESI treatment a routine qPCR [4] for *T. cruzi* in peripheral blood was repeated with a negative result, but after 1 year of follow-up and once treatment was stopped, a positive qPCR for *T. cruzi* was obtain. Thus, she received first line treatment for CD with benznidazole 5 mg/Kg/day for 60 days with good tolerability, with yearly *T. cruzi* qPCR in peripheral blood negative during 3 years of follow-up (Fig. 1).

## Discussion and conclusions

Several studies have been published using different ESI for CD treatment, initially against benznidazole resistant *T. cruzi* strains. Ketoconazole demonstrated in vitro activity against *T. cruzi* but failed to induce cure in patients with chronic phase of the disease [5]. Other ergosterol inhibitors were then tested and Venegas et al. demonstrated the effectivity of itraconazole on 20 patients with the negativization of the peripheral parasitemia in 50% of the patients using xenodiagnoses on previously positive patients and the decrease of sera lytic activity on the responder patients [6]. Urbina et al. found better cure rates with posaconazole in acute and chronic murine models compared to ketoconazole [4]. In the same line, Molina et al. tested posaconazole in a murine model of acute and chronic infection with cure rates of 90 and 60% respectively, superior than those treated with benznidazole even in immunosuppressed mice [7]. In humans, case reports of successful treatment with the triazole derivative posaconazole, have been published, even after benznidazole treatment failure in immunosuppressed patients [8]. Hence, different clinical trials had been carried out to evaluate the efficacy and safety of different triazole derivatives. In the CHAGASAZOL trial, posaconazole at different doses was compared to benznidazole in peripheral blood positive qPCR patients. Even when patients received the maximum dose approved for human use, in the intention-to-treat analysis, posaconazole showed higher treatment failure rates measured by peripheral blood qPCR of as much as 80.7% compared to a 38.4% obtained with benznidazole [9]. To come up with an optimization of treatments, after some promising combination studies performed in mice [10], the STOP-CHAGAS trial compared benznidazole and posaconazole in monotherapy and in combination. Benznidazole in monotherapy showed a parasitological cure of 86.7%, higher than posaconazole in monotherapy (13.3%) and even higher than both treatments in combination (80%) [3]. Later on, a ravuconazole prodrug, which had a better biodisponibility and half-live, was also tested in a clinical trial. In that study, treatment with different doses of ravuconazole was compared to standard treatment with benznidazole. After a 12-month follow-up, only 29% of patients treated with high-dose ravuconazol had sustained parasite response compared to 82% in patients treated with benznidazole [11]. Recently, different studies testing ESI in mice using novel test of cure as the bioluminescence, showed poor results. Francisco et al. performed a chronic phase murine model whereas benznidazole showed a 100% of parasitological cure, posaconazole failed in almost all cases. These inferior cure rates were also observed when tested in the acute model when new cure standard were applied [12]. Different mechanisms were proposed to explain these results. In the late chronic stage of CD*, T. cruzi* may have quiescent amastigote forms with low replicative rates, therefore treatment using ESI, which also acts during the replicative phase, could not be enough to eliminate the parasite [13]. Additionally, it has been stated that the dose of posaconazole used in both clinical trials, although being the maximum approved in humans, would suppose a drug exposure of 10 to 20% of the cure dose administered in mice [14]. Hence, longer treatments with ESI have been suggested to overcome that lack of efficacy. The patient presented here received twelve months of itraconazole to treat an ileal histoplasma infection. However, erratic drug absorption and distribution could have occurred since drug levels during treatment were not obtained. Although little evidence is derived from a single case, our patient showed a positive qPCR persistence in peripheral blood at the end of treatment, which is in line with a previous case published by Galhardo et al. [15] and previous murine models suggesting scarce benefit of prolonged ESI treatment [16]. In our opinion, despite the contradictory evidence of ESI in the animal model, all published data including our case suggests that ESI failure rates in positive peripheral blood qPCR are higher than that obtained with the current treatments of choice. Due to the fact that HIV patients are at risk of reactivation, prompt etiological treatment and close follow-up should be guaranteed.

## Data Availability

Data sharing is not applicable to this article as no datasets were generated or analysed during the current study.

## Abbreviations

CD: Chagas disease
ESI: Ergosterol synthesis inhibitors
qPCR: real-time quantitative polymerase-chain-reaction
